# Patterns of Injury in Hospitalised One-Year-Old Children: Analysis by Trimester of Age Using Coded Data and Textual Description

**DOI:** 10.3390/ijerph13070674

**Published:** 2016-07-07

**Authors:** Debbie Scott, Victor Siskind

**Affiliations:** 1 Australian Institute of Family Studies, Melbourne, Victoria 3000, Australia; debbie.scott@aifs.gov.au; 2 Centre for Accident Research and Road Safety, Queensland 4059, Australia

**Keywords:** injury, injury surveillance, child development, injury prevention

## Abstract

The second year of life is a time of rapid developmental changes. This paper aims to describe the pattern of unintentional injuries to one-year old children in three-month age bands to better understand the risks associated with developmental stages and, therefore, identify opportunities for proactive prevention. Injury surveillance data were used to identify children admitted to hospital in Queensland, Australia for an unintentional injury from 2002–2012. Falls were the most common injury, followed by burns and scalds, contact injuries and poisonings. Falls and contact injuries remained roughly constant by age, burns and scalds decreased and poisonings (by medications) increased. Animal- and transport-related injuries also became more common, immersions and other threats to breathing less common. Within the falls and contact categories falls from play equipment and injuries due to contact with persons increased, while falls down stairs and catching fingers in doors decreased. The pattern of injuries varies over the second year of life and is clearly linked to the child’s increasing mobility and boldness. Preventive measures for young children need to be designed—and evaluated—with their developmental stage in mind, using a variety of strategies, including opportunistic, developmentally specific education of parents; and practitioners should also consider potential for lapses in supervision and possible intentional injury in all injury assessments.

## 1. Introduction

Injuries to young children can have significant immediate and long-term adverse health and emotional effects, yet are largely potentially avoidable [1,2,3]. The second year of life is a time of rapid developmental change, and development is likely to play a significant role in the type and severity of injuries. Identification of injury intent in this age group can be difficult to ascertain, particularly in fatal injury [4]. Previous analyses of emergency department injury presentations in children found that 22% linked to a child protection record within six-months of the injury presentation [5]. Knowledge of the circumstances of such injuries can guide efforts to prevent unintentional injuries, minimize their effects and possibly assist in informing circumstances of child maltreatment related injury or lapses in supervision that lead to injury through recognition of injury patterns that do not conform with the developmental stage and ability of the child. 

Most published analyses of injuries in children over one year and under five years of age do not examine them in single years, whereas this is a period of life when growth and development are so rapid as to necessitate analysis in far narrower age bands. 

Few papers analyze injury patterns in such narrow age bands. A recent article published by New South Wales researchers identifies the leading causes of injury in children under 5 years of age hospitalized in that state over the years 1999 to 2009 by single year of age and gives age-specific rates [6]. Apart from this, there appears to be only one paper in English which describes injury profiles in one-year-old children in three months intervals, an analysis of California hospital and death certificate records over the years 1996 to 1998 [7]. 

We recently published a paper describing the pattern of injuries in hospitalized infants under one year by trimester of age using data collected and supplied by the Queensland Injury Surveillance Unit (QISU) for the years 2002 to 2007 [8]. This pattern altered appreciably over the age interval, influenced by the rapid growth and development of children in their first year. We have undertaken a similar examination for children in the second year of life when growth and development are still rapid, though somewhat less so than in younger infants.

The categorization of young children by year of age is entirely arbitrary, for the convenience of statistical tabulation rather than for any intrinsic validity. We present below the distribution of causes of injury in children aged under two years in narrower age bands, showing that the largest changes occur at nine months rather than one year, corresponding to the greatly increased mobility which occurs in children at the former age.

In the current paper we describe the pattern and causes of injury to one-year-old children admitted to hospital by trimester of age using coded data and textual description and make some comparisons with the patterns in younger children.

## 2. Methods

This paper analyses injury surveillance data collected between 2002 and 2012 by the Queensland Injury Surveillance Unit (QISU). For the one-year-old children we used the same data file supplied by QISU for the years 2002 to 2007 and identical methods as for the infants under a year, supplemented by a similar but more recent file provided by QISU, covering the years 2008 to 2012. The scope of and procedures used by QISU are described in detail in a previous paper [8]. Briefly, QISU collects injury data from a convenience sample of Queensland public hospital emergency departments. Triage nurses collect the data at the initial patient presentation for the treatment of an injury as part of the emergency department admission process. Items collected include external cause, principal diagnosis, major injury factor, mechanism of injury and destination after discharge from the emergency department, all in coded form, as well as patient demographics, for small children only age and sex, and a narrative text descriptor of the presenting problem which often includes a description of the injury event. No other socio-demographic information is collected. 

A certain degree of ambiguous classification of the injury is to be expected, despite the significant training provided to triage nurses who collect the data, due to the usual circumstances in an emergency department. Therefore, a large element of the study involved careful reading the brief textual description which accompanies each record in the data file to glean information not readily available in the coded entries. Only children who were admitted to hospital for their injury were included in this analysis (18% of all records in this group), consistent with our previous paper [8] and because these injuries likely represent the more severe injuries. Only injuries specifically coded as unintentional (accidental) are included because unintentional and intentional (inflicted) injuries in children are quite different [9] and, while many of the risk factors are shared, the prevention of intentional injury requires different strategies than for unintentional injury. Obvious allergic reactions, epileptic seizures leading to injury, and hemophiliac bleeding without apparent injury were also excluded.

Injuries were analyzed in terms of external cause at any age from 12 to 23 months and in three-month (trimester) groupings. In the great majority of cases the coded cause was used in the classification, but where obvious errors and ambiguities were detected from an examination of the coded text, the injury was reclassified. Furthermore, injuries in the categories, Other Specified Cause and Unspecified Cause were re-assigned to specific categories wherever possible by examination of the text description and other relevant data items.

As indicated below, the age distribution of hospitalized children in the one-year age group is flat and symmetric, implying that trends by age in the various categories could be tested by standard analysis of variance techniques. Differences in distributions were tested by standard contingency table methods.

## 3. Results

After the exclusions mentioned above a sample of 3585 one-year-olds was left, 57.0% male. In contrast to the infants, where the number of admitted children in the sample doubled from the first trimester of age to the fourth [8], the numbers of one-year-olds in the sample was almost constant in each trimester of age, ranging from 828 in the first trimester (23.1%) to 976 in the second (27.2%). Eighty-five percent of these children were admitted, often after transfer, to one or other of the two dedicated children’s hospitals in Brisbane at that time. The proportion of males does not change appreciably over the year and there are no significant differences between the sexes in external cause, nature of injury or body region affected. 

The distribution of external causes of injury by trimester is shown in Table 1. As with infants the major contributors to injury are falls, burns and scalds, contact and poisons. Together these four account for 83.1% of injuries in this age group, compared to 88.7% in infants under a year. Falls, contact, cutting/piercing, foreign bodies and other or unspecified injuries remained fairly constant absolutely and as a proportion over the year, without evidence of a trend. Poisonings, transport-related injuries and animal-related injuries increased over the year, poisonings and transport-related injuries significantly so. Burns and scalds, and other threats to breathing other than by immersion (mainly choking on food and other inhaled or ingested objects) decreased significantly, while the decrease in immersions was not statistically significant.

As foreshadowed above, Table 2 shows how the pattern of injuries in children under two years of age developed with age, with a marked change at about nine months, with the injury profile of children in their fourth trimester becoming more similar to that of children in the next older trimester.

Almost 1400 injuries were due to falls, too many to allow scrutiny of every descriptive text. Only the descriptions for the fifth (12–15 months of age) and eighth (21–24 months of age) trimesters, 289 and 328 records respectively, were read. This allowed for a comparison of falls at the beginning and the end of the time period and provided sufficient detail for comparison. The fall data were recoded to a number of sub-categories, as shown in Table 3. The distributions differed significantly; in particular, there were noteworthy increases in falls from play equipment and high places other than furniture and decreases in falls down stairs, from high chairs and being dropped by a family member.

As in the case of infants, the head was the body region most likely to be injured in a fall, with 80% of falls resulting in head injuries, somewhat less than among infants. No age trend in the proportion of head injuries was evident. A different classification of injury nature was used in the later data to that in the earlier data, with a more specific category of head injury, intracranial injury, being introduced. In the 2008–2012 data file, the prevalence of reported intracranial injury among one-year-olds who fell was 17.2% very similar to that among children under one year of age (16.5%).

Burns and scalds decreased absolutely and relatively with age over the year. The breakdown by causative agent is given in Table 4. Hot liquids and household appliances, including the household iron, were by far the most common agents, together accounting for 78.5% of such injuries. In thirteen instances the scalds were due to excessively hot water from the household system, suggesting that the recommendation to decrease the temperature of the hot water supply to a safer level had not been followed in all households at that time. The distribution of agents showed no evidence of an age trend.

Injuries due to poisonings increased markedly absolutely and relatively with age, doubling from the first six months to the last three. The agents fell into five categories, the main ones being medications, comprising 45% of poisonings, and household chemicals (mainly cleaning products, bleach, pesticides and turpentine) making up a further 36%. Essential oils (natural oils extracted from plants with the characteristic odor of the plant, e.g., lavender, eucalyptus) formed a separate category comprising 8% of poisonings, with other chemicals and other substances, mainly plant material, making up the remainder (Table 5). Poisonings due to medications and to essential oils increased significantly with age over the year; poisonings in the other categories decreased correspondingly as a proportion, while remaining more or less constant in absolute numbers. 

Injuries classified under the category, contact, remained more or less constant from trimester to trimester. Those caused by a person rose significantly from about 8% in the first two trimesters to about 20% in the second two. Much of this increase was due to injuries, mainly dislocations, to the child’s arm through being pulled, often to prevent a fall, which formed overall 41% of injuries caused by a person. Over one third of contact-related injuries were due to the child catching fingers or a hand in a door or other object; this mode of injury decreased significantly with age over the twelve months (Table 6). 

For some injuries, the presenting problem text elaborated on the role of the parent, sibling or other family member in the injury. In infants 15.8% of injuries showed some involvement of family members [8], but in this sample of one-year-old children this proportion had decreased to 3.3% (data not shown).

## 4. Discussion

As was the case with younger children, the pattern of injuries in one-year olds continues to evolve over the course of the second year of life. Mobility and competence develop rapidly in children of this age and this is reflected in the changing ways in which children are injured at this stage of their lives. If preventive efforts are to be effective these changes should be recognized and prevention strategies tailored to developmental stages. Parents and caregivers should be provided with knowledge about child development and injury risk when they come into contact with health professionals or other service providers so as to anticipate and prevent, or minimize these injuries. 

While the incidence of some injury causes, such as falls, contact and foreign body injuries, remained more or less constant, others, notably burns and scalds, but also threats to breathing, decreased or, like poisonings, increased. Even when the overall incidence of an injury class did not alter substantially, within that class there were some changes with age in the modes of injury; for instance, falls from play equipment and from high places other than furniture became more common, while falls down stairs or being dropped by someone became rarer. Similarly in the injury category of contact, having a hand caught in a door or elsewhere became less common with age while injuries due to other persons became more common.

The incidence of poisonings increased markedly with age among children aged one year, and this is consistent with the findings of a study of hospital admissions in another state [10]. In our data the increase was entirely due to the ingestion of medications and essential oils, with poisonings from other sources remaining more or less constant in absolute number. While some text descriptions of the injury event reported that some children climbed on furniture to reach medications on tables or kitchen counters or medications were knocked down by older siblings, unfortunately the brevity of most of the text descriptions did not permit a detailed examination of the ways in which the children gained access to the medications they swallowed. At least one injury was described as having occurred because an older sibling administered a medication stored in the fridge door to a younger sibling. These data also suggest that ingested medications were not in secure containers. The Australian Standard 1928 [11] requires performance testing of closures and requires that not more than 15% of a panel of 200 children between 42 and 51 months be able to open the closure. Additionally the standard requires that no more than 10% of a panel of 100 adults between 18 and 55 years be unable to open and re-engage the closure. Effectively this means that some children will always be able to open child resistant packaging and some adults will always find these closures difficult to access. Accordingly, there is a possibility that containers in regular use are not properly sealed after every opening Some support for this suggestion is provided by a study of childhood poisonings due to over-the-counter medications that found that where medications had been in a child-resistant container, in almost half of instances the container had not been properly secured after last use [12]. Parents and caregivers may not consider or realize that essential oils are as harmful as medications; they may not be sold in childproof packaging, may not always be stored in a safe place or toddlers may access them from easily accessible handbags of visitors or even from shopping bags before contents are put away and moved to safe storage places. The exact ways in which toddlers access and are poisoned by medications or essential oils warrants detailed examination in its own right.

Although there are clear differences in some injuries in these data, details of caregiver supervision are not available to assist in interpreting the data. Injury patterns may change as parents and/or caregivers recognize that, with increasing mobility and a desire to explore their environment, children’s behaviors, and hence risks they are exposed to, are changing. Parental behaviors may be modified as they recognize this change in their children and this may contribute to changing injury patterns, though the concept of “adequate supervision” remains ambiguous and is not well-defined in the literature [13]. There is research demonstrating that children who have histories of child maltreatment are twice as likely to die of an unintentional injury than children who are not previously known to child protection authorities [4]. Studies investigating fatal neglect have found that one-off episodes of supervisory lapses are common [14,15].

Several studies have pointed out that injury causation in toddlers is often the result of interplay between familial, circumstantial and individual factors [16,17]. Similar risk factors have been identified in the child protection literature as associated with child maltreatment, particularly in association with neglect and supervisory neglect. Parents who have poor mental health and substance misuse issues as well as larger families and those families where domestic and/or interpersonal violence are problems are more likely to be associated with neglectful parenting behaviors, including poor supervision or a lack of awareness of risk for their children [18,19,20] and, consequently an increased likelihood of injury. 

In a workshop on child injury prevention in Canada in 2013 several experts on child injury pointed out that over-emphasis on keeping small children safe may result in compromising healthy development [21]. In particular one participant (Dr. Sandseter from Norway) distinguished between hazardous childhood activities (to be avoided) and risk-taking (crucial to normal child development). Curtailing the latter, she claimed, could adversely affect a child’s ability to “learn risk perception and management skills important in developing understanding of how to ... avoid injuries”. Other participants commented that a similar effect might ensue from designing play areas with excessive concentration on safety rather than challenge. Such considerations are necessarily absent from workplace and road safety thinking, but should perhaps play a greater role in designing injury prevention interventions for small children. It should however be noted that this point of view has not gone unchallenged [22,23].

In respect of possible interventions, it is clear that regular reminders to care-givers about the importance of keeping hot or hazardous substances out of the reach of small children is fundamental, as is safety in and around motor vehicles and near stairs. It is however worth noting that a recent systematic overview of the evidence of the effectiveness of interventions to prevent scalds in children found little to show for such interventions in reducing the incidence of scalds in children [24,25], suggesting that the design of such interventions is challenging. With regard to other modes of injury the position is more complex. 

### Limitations

As discussed in our previous paper, the sample is based on a relatively limited number of participating Queensland hospitals and may not be entirely representative of all injured children in the State. However 85% of the sample were treated in one or other of the two dedicated children’s hospitals in the capital city at the time of the study, often after transfer from other hospitals. Information on the injury background of transferred children is sometimes sketchy, and in a busy emergency department data collection and coding may on occasion be imperfectly done. Nonetheless, as we reported in the previous paper, a prospective study has found data collected in this way to be reliable as an estimator of incidence and severity [26].

## 5. Conclusions

Reporting injury patterns in very young children even by single year of age tends to obscure the significant changes in injury profiles which occur as children go through a period of rapid growth and development in the first two years of life and possibly longer. Understanding the way the patterns alter with age can better inform interventions targeted specifically to the various phases of childhood. Where parents have information about the injury risks associated with increased mobility and independence of children they are better able to identify and minimize exposure to risk and prevent what may me a life threatening or limiting injury. Wider dissemination of this understanding to parents, caregivers and health professionals will enable them to anticipate the sort of injuries that children in their care may incur and improve child safety, regardless of the type or intent of injury.

## Figures and Tables

**Table 1:** Distribution of external causes of injury in children admitted to hospital aged one year, by trimester of age.

External Cause Category	Frequency (%) by Trimester					
	5th	6th	7th	8th	Total	%
Falls	289 (34.9)	385 (39.4)	383 (41.1)	328 (38.7)	1385	38.6
Burns & Scalds	202 (24.4)	233 (23.9)	166 (17.8)	125 (14.7)	726	20.3
Contact or collision with	109 (13.2)	130 (13.3)	139 (14.9)	114 (13.4)	492	13.8
Poisons	69 (8.3)	68 (7.0)	103 (11.0)	136 (16.0)	376	10.5
Animal related	20 (2.4)	33 (3.4)	32 (3.4)	32 (3.8)	117	3.3
Foreign Body	39 (4.7)	23 (2.4)	19 (2.0)	36 (4.2)	117	3.3
Transport	16 (1.9)	34 (3.4)	25 (2.7)	34 (4.0)	109	3.0
Cutting/piercing	26 (3.1)	23 (2.4)	27 (2.9)	20 (2.4)	96	2.7
Immersion	13 (1.6)	18 (1.8)	13 (1.4)	5 (0.6)	49	1.4
Other threats to breathing	26 (3.1)	16 (1.6)	19 (2.0)	11 (1.3)	72	2.0
Other & unspecified	19 (2.3)	13 (1.3)	7 (0.8)	7 (0.8)	46	1.3
Total	828 (23.1)	976 (27.2)	933 (26.0)	848 (23.7)	3585	

F (10, 3574) = 7.44, *p* < 0.001.

**Table 2:** Distribution (%) of the cause of injury in the first two years of life.

Age in Months	0–8	9–11	12–14	15–23
N	660	398	828	2757
Falls	65.4	38.9	34.9	39.6
Burns & scalds	11.6	22.4	24.4	19.0
Contact or collision with	8.7	11.3	11.3	18.8
Poisoning	3.1	7.9	8.4	11.2
Other	11.2	19.5	19.1	20.7

**Table 3:** Types of falls occurring in the first and fourth trimesters of age in children aged one-year admitted to hospital.

Type of Fall	12 to 14 Months		21 to 23 Months	
	*n*	%	*n*	%
“Fell”	84	29.1	82	25.0
From bed	20	6.9	30	9.1
From other furniture	42	14.5	60	18.3
From a another high place	20	6.9	39	11.9
Down stairs	51	17.6	24	7.3
From a high chair	11	3.8	5	1.5
From play equipment	12	4.2	40	12.2
Dropped by a family member	12	4.2	5	1.5
Other and unspecified	37	12.8	43	13.1
Total	289	34.9	328	38.7

χ^2^ (d.f. = 8) = 39.4, *p* < 0.0001.

**Table 4:** Agents causing burn or scald injuries in children aged one year admitted to hospital.

Agent	*n*	%
Hot beverage	256	35.3
Hot water	185	25.5
Household heating/cooking appliance	129	17.8
*Iron* *	*48*	*6.6*
Hot food or oil	30	4.1
Other hot objects or hot surfaces	49	6.7
Fire/coals/ashes	37	5.1
Chemicals incl. acid	9	1.2
Other & unspecified	31	4.3
Total	726	100.0

* Included in Household heating/cooking appliance total.

**Table 5:** Agents involved in poisonings of children aged one year admitted to hospital.

Agent	Frequency (%) by Trimester					
	5th	6th	7th	8th	Total	%
Medications	18 (26.1)	22 (32.4)	53 (51.5)	76 (55.9)	169	44.9
Essential oils	6 (8.7)	3 (4.4)	5 (4.9)	16 (11.8)	30	8.0
Household chemicals	36 (52.2)	35 (51.5)	35 (34.0)	31 (22.8)	137	36.4
Other chemicals	3 (4.3)	4 (5.9)	6 (5.8)	7 (5.1)	20	5.3
Other & unspecified	6 (8.7)	4 (5.9)	4 (3.9)	6 (4.4)	20	5.3
Total	69 (18.4)	68 (18.1)	103 (27.4)	136 (36.2)	376	100

χ^2^ (d.f. = 12) = 35.66, *p* < 0.001.

**Table 6:** Causes of injury under the Contact category.

Injury Cause	Frequency (%) by Trimester					
	5th	6th	7th	8th	Total	%
Finger/hand caught in object	54 (49.5)	61 (46.9)	40 (28.8)	33 (28.9)	188	38.2
In door *	*38 (34.9)*	*46 (35.4)*	*29 (20.9)*	*24 (21.1)*	*137*	*27.8*
Object struck or fell on child	20 (18.3)	20 (15.4)	31 (22.3)	21 (18.4)	92	18.7
Ceiling fan *	*4 (3.7)*	*3 (2.3)*	*4 (2.9)*	*3 (2.6)*	*14*	*2.8*
Caused by person ^1^	9 (8.3)	11 (8.5)	25 (18.0)	23 (20.2)	68	13.8
Arm injured by pulling *	*1 (0.9)*	*3 (2.3)*	*12 (8.6)*	*12 (10.5)*	*28*	*5.7*
Child collided with object	11 (10.1)	21 (16.2)	28 (20.1)	16 (14.0)	76	15.4
Other/unspecified	15 (13.8)	17 (13.1)	15 (10.8)	21 (18.4)	68	13.8
Total	109 (22.2)	130 (26.4)	139 (28.3)	114 (23.2)	492	

* additional detail relating to cause of injury above; ^1^ including other child. χ^2^ (d.f. = 12) = 30.52, *p* = 0.002.
